# Nonadherence to inhaled corticosteroids is associated with negative mepolizumab response

**DOI:** 10.1183/23120541.00135-2026

**Published:** 2026-10-19

**Authors:** Mohammed Ghazi Almutairi, John Marriott, Zahraa Jalal, Louise Wood, Samantha Caddick, Babita Rajkumari, Susan Renwick, Siobhan Wilkinson, Caroline Gallagher, Hollie Morgan, Adel H. Mansur

**Affiliations:** 1 Respiratory Department, Heartlands Birmingham University Hospitals NHS Trust, Birmingham, UK; 2 Department of Pharmacy Practice, College of Pharmacy, Qassim University, Buraydah, Saudi Arabia; 3 Pharmacology School, University of Birmingham, Birmingham, UK; 4 Institute of Inflammation and Ageing, University of Birmingham, Birmingham, UK

## Abstract

**Background:**

Nonadherence to inhaled corticosteroids (ICS) in severe asthma is associated with poor control and may impair response to biologics. The primary objective of the present study was to evaluate ICS nonadherence influence on mepolizumab treatment response in adults with severe eosinophilic asthma.

**Methods:**

In this retrospective cohort study, we compared ICS nonadherence rates in mepolizumab responders and nonresponders. Adherence was determined using a prescription possession ratio (PPR) of ≥75% to indicate adherence. Mepolizumab response was assessed using the positive response criteria of a ≥50% reduction in exacerbations and/or in the maintenance oral corticosteroid dose. Predictors of adherence and mepolizumab response were explored using standard statistics.

**Results:**

A total of 190 participants were included in the analysis (enrolment mean±sd age 52±13 years, 59 (31%) males). There were 153 (80%) responders to mepolizumab who displayed lower median exacerbations than the nonresponders (1 (interquartile range (IQR) 0, 2) *versus* 4 (IQR 2, 7); p=0.002). ICS nonadherence was detected in 30% and 26% of participants prior to and during mepolizumab treatment respectively. ICS nonadherence was more common in nonresponders 12/23 (52%) than in mepolizumab responders 29/130 (22%), p=0.002. Nonadherent patients had higher exhaled nitric oxide fraction (*F*_ENO_) (ppb) than the adherent patients (median 57 (IQR 35, 81) *versus* 34 (IQR 21, 61); p=0.009). Pre-mepolizumab nonadherence correlated significantly with on-treatment nonadherence (r=0.75, 95% CI 0.67–0.81; p<0.0001).

**Conclusions:**

ICS nonadherence was associated with negative response to mepolizumab treatment and raised *F*_ENO_ levels compared to the adherent group. Our findings support the notion for the need for optimisation of ICS adherence during mepolizumab therapy.

## Shareable abstract

**ICS nonadherence is associated with negative mepolizumab response. Pre-biologic nonadherence was the main predictor of continuing ICS nonadherence during treatment. Optimisation of ICS adherence is recommended to maximise mepolizumab response.** https://bit.ly/4uuG1uI

## Introduction

Severe eosinophilic asthma patients (defined by high blood eosinophil or sputum eosinophilia) suffer from poor symptoms and risk of exacerbations despite high-dose inhaled corticosteroids (ICS) and other asthma medication. They often require rescue or maintenance oral corticosteroid (OCS) to manage their exacerbations [1]. Consequently, they suffer significant morbidity and risk of mortality owing to poor control or treatment-related side-effects [2].

Mepolizumab is a monoclonal antibody that binds the circulating interleukin (IL)-5 and blocks interactions with its receptor on the eosinophil cell surface, thereby reducing eosinophil production, maturation and activation [3]. Mepolizumab was approved as an add-on treatment for patients with severe eosinophilic asthma and significantly reduced exacerbations and improved asthma control, with up to 70% of patients reported achieving positive clinical response [4]. However, it is impossible to predict mepolizumab response without a treatment trial. Nonadherence to ICS is prevalent (≈40%) in severe asthma patients [5], and may negatively influence mepolizumab response. Adherence to ICS in patients on mepolizumab was reported to be associated with better symptom control, while nonadherence contributed to poor control [6, 7]. Although ICS adherence is a prerequisite to commencing biologics, the lack of accurate adherence measures means that adherence assessment is not always precise [8, 9]. Also, patients who improve on mepolizumab may be at a higher risk of becoming nonadherent, as a lack of symptoms would reduce the urge to take ICS regularly [10].

Exhaled nitric oxide fraction (*F*_ENO_) is an inflammatory biomarker produced by airway epithelium. *F*_ENO_ level increases in asthma, suppressed by ICS and a raised *F*_ENO_ level despite ICS use would indicate either nonadherence or ICS resistance [11, 12]. Raised *F*_ENO_ predicts future risk of exacerbations [13]. Mepolizumab has a modest effect on *F*_ENO_ as it primarily blocks the IL-5 axis, whilst *F*_ENO_ production is driven mainly through the IL-13 axis [14]. However, it is unknown if patients with persistently high *F*_ENO_ despite mepolizumab have a lower response rate than patients with suppressed *F*_ENO_. In well-controlled patients treated with benralizumab, raised *F*_ENO_ in the context of down-titration of ICS dose led to reduction in lung function and increased risk of loss of control [15]. Therefore, the above may argue for a link between lower ICS use, high *F*_ENO_, loss of asthma control and a negative response to biologics targeting IL-5 axis.

Understanding the drivers of unresponsiveness to mepolizumab may allow better strategies that can enhance the response rate. In this study, we explored the effect of ICS nonadherence on mepolizumab response and markers of ICS nonadherence to improve mepolizumab response rate by identifying a subgroup of patients that may benefit from interventions to improve adherence.

## Methods

### Study design

The study primary aim was to explore the effect of ICS adherence on mepolizumab treatment response and secondary aims were to explore predictors of both ICS adherence and mepolizumab treatment responses. We invited all adult patients (≥18 years) who completed 12 months of mepolizumab treatment at our centre from June 2017 to June 2023 to take part in this study. We collected data retrospectively from all those who consented to participate. Exclusions included those who did not complete 12 months of treatment and those who did not consent to participate. The study was approved by the Integrated Research Ethical Committee in UK (study number 214026) and registered on ClinicalTrials.gov (NCT05241769).

### Setting and participant selection

The study was conducted at the Birmingham Regional Severe Asthma Service (BRSAS). The BRSAS network covers the West Midlands, Derbyshire and Gloucestershire in Central England, with a catchment area of 7.3 million [16]. Patients were commenced on mepolizumab if they met the National Institute for Health and Care Excellence (NICE) criteria [17] for mepolizumab treatment initiation. Treatment delivered over 12 months and outcomes were measured used standardised proformas across the network that captured relevant asthma clinical outcomes. To reduce the risk of selection bias, all patients treated with mepolizumab during the specified time were identified and those who completed 12 months of treatment were approached to take part regardless of the treatment outcome status.

### Data sources and measurements

The 12 months before and after mepolizumab treatment start data were retrieved from standardised *pro formas* designed to provide systematic patient assessment and monitoring during mepolizumab treatment. The NICE criteria of treatment response [17] were applied to determine mepolizumab treatment response (*i.e.* ≥50% reduction in OCS requiring severe exacerbations and/or ≥50% reduction in maintenance OCS daily dose from baseline). Adherence to ICS treatment was determined by measuring the number of inhaler prescriptions collected over the 12 months before and during the 12 months of mepolizumab treatment. Prescription pick-up history was retrieved from general practitioners’ records and a prescription possession ratio (PPR) was calculated for each patient (number of monthly prescriptions collected in 12 months/12×100) with a ratio of ≤75% considered to indicate nonadherence [10].

ICS adherence determined by PPR in the responder and nonresponder groups were compared as dichotomous (cut-off PPR ≤75%) and continuous traits. Comparisons were also made using mepolizumab response status and ICS adherence for clinical outcomes that included exacerbations, maintenance OCS (yes/no and mean daily dose), ICS dose (μg·day^−1^), proportion with ≥1 emergency healthcare visits, ≥1 hospital admissions, ≥1 intensive therapy unit admissions, mean 6-item Asthma Control Questionnaire (ACQ-6) score and proportions with ACQ-6 score <1.5 (well-controlled), and lung function. Changes in *F*_ENO_ (ppb) and blood eosinophil count (n×10^9^·L^−1^) were explored at baseline *versus* 12 months of treatment and highest recorded in the 12 months pre- and post-treatment start. Comparisons were also conducted at *F*_ENO_ cut-off points of 25, 35 and 50 ppb, and blood eosinophils (0.15, 0.3, 0.5 and 0.7×10^9^·L^−1^). The proportion of cases achieving clinical remission following 12 months of mepolizumab treatment were determined using three criteria (not on maintenance OCS, no exacerbations and ACQ-6 score <1.5) [18].

### Statistical analyses power calculation

Statistical analyses were conducted to examine the primary hypothesis that ICS nonadherence reduces the treatment response to mepolizumab. In the sample size calculation, we assumed a 70% positive mepolizumab response rate and a nonadherence prevalence of 40% in the nonresponder group and 20% in the responder group. For this, a sample size of 187 participants comprising 70% (131) responders and 30% (56) nonresponders has 80% power to detect statistical significance at 0.05. The impact of ICS nonadherence on asthma outcomes such as *F*_ENO_ and blood eosinophils was explored. We also examined the clinical outcomes of the whole cohort and in the responder and nonresponder groups. We used a t-test for normally distributed continuous variables or the Mann–Whitney–Wilcoxon test for non-normally distributed variables. Cross tabulation and chi-square tests were conducted to compare dichotomous traits. Data were presented as means with standard deviations or as medians with interquartile ranges (IQRs) for normally and non-normally distributed data, respectively. Missing data for parameters were indicated in the results by providing numbers included in the analysis per each variable. Multivariate analyses were conducted to look at variables influencing ICS adherence in which we used ICS adherence as the dependent variable and age, body mass index (BMI), baseline ICS adherence, asthma exacerbations, accident and emergency unit (A&E) attendances, ACQ-6 score, eosinophil count and *F*_ENO_ as the independent variables. Multiple regression analyses were also conducted to explore predictors of mepolizumab response. Data analyses were conducted using Stata/SE 16.1 software for Mac (Stata Statistical Software, StataCorp, 2020).

## Results

### Cohort clinical characteristics

A total of 422 eligible participants were approached, of which 201 (48%) consented to participate in the study and 190 (45%) had completed 12 months mepolizumab treatment with available treatment response data and were included in the analysis. There were no other exclusions applied to the study analysis sample. The clinical characteristics of participants included in the study are provided in table 1. The mean±sd age at enrolment was 52±13 years; 31% were male, 78% were White and 52% were in full-time employment. The mean±sd age of asthma onset was 24±18 years and 44% had early-onset asthma (<18 years). The median severe asthma exacerbation frequency was 6 (IQR 4, 8) per annum and 49% (94/190) were on maintenance OCS. In addition to asthma, participants had significant comorbidities that included obesity (50%), gastro-oesophageal reflux disease (GORD) (41.1%) and chronic rhinosinusitis (34.2%).

**TABLE 1:** Patient characteristics (n=190)

Parameter	Outcome
**Age at enrolment (years), mean±sd**	52±13
**Age ≥18–64 years, n (%)**	136 (72)
**Age ≥65 years, n (%)**	54 (28)
**Age at asthma diagnosis (years), mean±sd**	24±18
**Duration of asthma (years), mean±sd**	29.9±19.7
**Female, n (%)**	131 (69)
**Ethnicity, n (%)**	
White	148 (78)
Ethnic minorities	42 (22)
**Smoking history, n (%)**	
Never	95 (50)
Former	48 (25)
Current	6 (3.2)
Unknown	41 (21.6)
**BMI (kg·m^−2^), mean±sd**	32±8
**Comorbidities, n (%)**	
GORD	78 (41)
Chronic rhinosinusitis	65 (34)
Atopic dermatitis	57 (30)
Infective phenotype	52 (27)
Allergies	50 (26)
Osteopenia/osteoporosis	36 (19)
CVD	33 (17)
Diabetes	26 (14)
Sleep apnoea	24 (13)
Nasal polyposis	24 (13)
**Previous biologic (omalizumab), n (%)**	29 (15)
**ICS dose (mg·day^−1^), mean±sd**	808.7±432.6
**mOCS yes/no, n (%)**	94 (49%)
**ACQ-6 score, mean±sd**	3.06±1.19
**FEV_1_ (L), mean±sd**	1.9±0.75
**FEV_1_ (% pred), mean±sd**	73.04±29.44
**FVC (L), mean±sd**	2.8±0.91
**FVC (% pred), mean±sd**	84.29±22.7
**FEV_1_/FVC ratio, mean±sd**	66.1±14.3

ACQ-6: 6-item Asthma Control Questionnaire; BMI: body mass index; CVD: cardiovascular disease; FEV_1_: forced expiratory volume in 1 s; FVC: forced vital capacity; GORD: gastro-oesophageal reflux disease; ICS: inhaled corticosteroids; mOCS: maintenance oral corticosteroids.

### Whole cohort mepolizumab treatment outcomes

Treatment outcomes are presented in table 2. A positive response was observed in 153/190 (80%) participants. Of the remaining 37, 28 (15%) had a negative response and nine (5%) withdrew treatment due to other factors (*e.g.* side-effects). Clinical remission, defined as not receiving maintenance OCS (mOCS), having no exacerbations and an ACQ-6 score <1.5, was observed in 23/172 (13%). The median annual number of exacerbations for the whole cohort was reduced from 6 (IQR 4, 8) to 1 (IQR 0, 3), p<0.001. The proportion of participants on mOCS was reduced from 49% (94/190) to 41% (78/190), p<0.001. The ICS dose (µg·day^−1^) remained the same (pre 800 (IQR 400, 1000) *versus* end 800 (IQR 400, 1000), p=0.9), as did the ICS nonadherence rate (pre 48/160 (30%) *versus* end 42/160 (26%). There was a significant improvement in symptom control, with the proportion having an ACQ-6 score <1.5 increasing from 11% (18/170) to 38% (64/170), p<0.001. Median blood eosinophils (×10^9^·L^−1^) decreased from 0.53 (IQR 0.37, 0.80) at baseline to 0.07 (IQR 0.04, 0.11), p<0.001, at the end of treatment, but median *F*_ENO_ (ppb) did not change significantly (baseline 48 (IQR 22, 77) *versus* end 40 (IQR 22, 75) p=0.1). For those with available lung function data (n=125), FEV_1_ % pred improved from 73% pred (IQR 58, 87) to 77% pred (IQR 57, 93) at the end of treatment, p=0.005.

**TABLE 2:** Whole cohort outcomes following 12 months of mepolizumab treatment

Clinical measurement	Total number	Before mepolizumab	After mepolizumab	p-value
**Exacerbations, median (IQR)**	190	6 (4, 8)	1 (0, 3)	p<0.001
**On maintenance OCS, n (%)**	190	94 (49)	78 (41)	p<0.001
**Prednisolone dose (mg·day^−1^), median (IQR)**	78	10 (10, 20)	10 (5, 20)	p=0.3
**ICS dose (µg·day^−1^), median (IQR)**	184	800 (400, 1000)	800 (400, 1000)	p=0.9
**ICS nonadherence (PPR ≤75%), n (%)**	160	48 (30)	42 (26)	p=0.5
**Proportion with ≥1 A&E visit, n (%)**	190	45 (24)	18 (9)	p<0.001
**Proportion with ≥1 hospital admission, n (%)**	190	24 (13)	21 (11)	p=0.5
**Proportion with ≥1 ITU/HDU admission, n (%)**	190	5 (3)	2 (1)	p=0.2
**Proportion with (ACQ-6 score <1.5), n (%)**	170	18 (11)	64 (38)	p<0.001
**ACQ-6 score, mean±sd**	170	3.1±1.2	2.1±1.3	p<0.001
***F*_ENO_ (ppb), median (IQR)**	166	48 (22, 77)	40 (22, 75)	p=0.1
***F*_ENO_ highest (ppb), median (IQR)**	166	51 (25, 87)	60 (32, 93)	p=0.002
**Blood eosinophils (×10^9^·L^−1^), median (IQR)**	187	0.53 (0.37, 0.80)	0.07 (0.04, 0.11)	p<0.001
**Blood eosinophils (×10^9^·L^−1^), median (IQR)**	187	0.56 (0.4, 0.84)	0.11 (0.07, 0.17)	p<0.001
**FEV_1_ (% pred), median (IQR)**	125	73 (58, 87)	77 (57, 93)	p=0.005

A&E: accident and emergency unit; ACQ-6: 6-item Asthma Control Questionnaire; *F*_ENO_: exhaled nitric oxide fraction; FEV_1_: forced expiratory volume in 1 s; ICS: inhaled corticosteroid; IQR: interquartile range; ITU/HDU: intensive therapy unit or high dependency unit; OCS: oral corticosteroids; PPR: prescription possession ratio.

### Comparisons of mepolizumab responder and nonresponder groups

Clinical comparisons of the positive and negative response groups are presented in table 3. Response rates were higher in females and responders were older in age and were more likely to be adherent to ICS than the nonresponders. The responder group achieved significantly lower median exacerbations (1 (IQR 0, 2) *versus* 4 (IQR 2, 7), p=0.002), lower mean±sd ACQ-6 scores (1.85±1.24 *versus* 3.07±1.08, p<0.0001), lower median blood eosinophils (×10^9^·L^−1^) (0.07 (IQR 0.04, 0.10) *versus* 0.11 (IQR 0.05, 0.15), p=0.04) and lower *F*_ENO_ (statistically nonsignificant) (37 (IQR 21, 74) *versus* 51 (27, 83), p=0.3), than the nonresponder group. There was also a nonsignificant trend towards a lower proportion of the responder participants to be in the high *F*_ENO_ groups at cut-offs ≥25, 35 and 50 ppb than the nonresponders. There was also a nonsignificantly higher FEV_1_ % pred in the responder group than the nonresponder group.

**TABLE 3:** Comparisons of the outcomes of the responder and nonresponder groups after 12 months of mepolizumab treatment

Parameter	Total number	Positive responders	Negative responders	p-value
**Mepolizumab response**	181	153	28	–
**Age (years), mean±sd**	181	57±12.7	51±15.9	0.026
**Sex**	181			
Female, n (%)	125	114 (91.2)	11 (8.8)	0.0002
Male, n (%)	56	39 (69.6)	17 (29.1)	
**Exacerbations, median (IQR)**	176	1 (0, 2)	4 (2, 7)	0.002
**ACQ-6 score, mean±sd**	166	1.85±1.2	3.07±1.1	<0.0001
**Blood eosinophils (×10^9^·L^−1^) end, median (IQR)**	178	0.07 (0.04, 0.10)	0.11 (0.05, 0.15)	0.04
**Blood eosinophils (×10^9^·L^−1^) highest, median (IQR)**	178	0.11 (0.07, 0.16)	0.15 (0.09, 0.26)	<0.001
***F*_ENO_ (ppb) end, median (IQR)**	166	37 (21, 74)	51 (27, 83)	0.3
***F*_ENO_ (ppb) highest, median (IQR)**	169	61 (33, 94)	67 (30, 98)	1
***F*_ENO_ ≥25 ppb, n (%)**	169	97/144 (67)	19/25 (76)	0.4
***F*_ENO_ ≥35 ppb, n (%)**	169	76/144 (53)	16/25 (64)	0.3
***F*_ENO_ ≥50 ppb, n (%)**	169	53/144 (37)	13/25 (52)	0.1
**PPR (≤75%) on mepolizumab, n (%)**	153	29/130 (22.3)	12/23 (52.2)	0.002
**FEV_1_ (% pred), median (IQR)**	125	81.0 (57.0, 93.8)	67.5 (55.0, 95.7)	0.7

End of or during the 12 months mepolizumab treatment values are presented as means or medians depending on data distribution. ACQ-6: 6-item Asthma Control Questionnaire; *F*_ENO_: exhaled nitric oxide fraction; FEV_1_: forced expiratory volume in 1 s; IQR: interquartile range; PPR: prescription possession ratio.

### Effect of ICS adherence on mepolizumab treatment outcomes

Comparisons of adherent and nonadherent groups outcomes with available data following mepolizumab treatment are provided in table 4. ICS adherence data in the context of mepolizumab treatment response were available for 153 patients (130 responders and 23 nonresponders). ICS nonadherence was significantly more common in the nonresponder group 52% (12/23) than the responder group 22% (29/130), p=0.002 (figure 1).

**TABLE 4:** Asthma biomarkers and remission achievement in inhaled corticosteroid adherent and nonadherent groups

Parameter	Total number	Adherent	Nonadherent	p-value
**Number included, n (%)**	158	117 (74.1)	41 (26)	
**Most recent blood eosinophil (×10^9^·L^−1^), median (IQR)**	158	0.07 (0.05, 0.10)	0.09 (0.03, 0.13)	p=0.4
**Highest eosinophil (×10^9^·L^−1^), median (IQR)**	158	0.11 (0.07, 0.16)	0.13 (0.06, 0.17)	p=0.9
**Most recent *F*_ENO_ (ppb), median (IQR)**	126	34 (21, 61)	57 (35, 81)	p=0.009
**Highest *F*_ENO_ (ppb), median (IQR)**	149	53 (26, 88)	77 (51, 118)	p=0.004
***F*_ENO_ ≥25 ppb, n (%)**	149	75/110 (68)	30/39 (77)	p=0.3
***F*_ENO_ ≥35 ppb, n (%)**	149	61/110 (55)	22/39 (56)	p=0.9
***F*_ENO_ ≥50 ppb, n (%)**	149	44/110 (40)	15/39 (38)	p=0.9
**Achieved clinical remission, n (%)**	9	6/9 (67)	3/9 (33)	p=0.9
**FEV_1_ (% pred), median (IQR)**	98	83.2 (57.5, 96.0)	75.0 (49.0, 85.8)	p=0.12
**Exacerbations per annum**	156	1 (0, 2)	1 (0, 3.8)	p=0.38

*F*_ENO_: exhaled nitric oxide fraction; FEV_1_: forced expiratory volume in 1 s; IQR: interquartile range.

**FIGURE 1:**
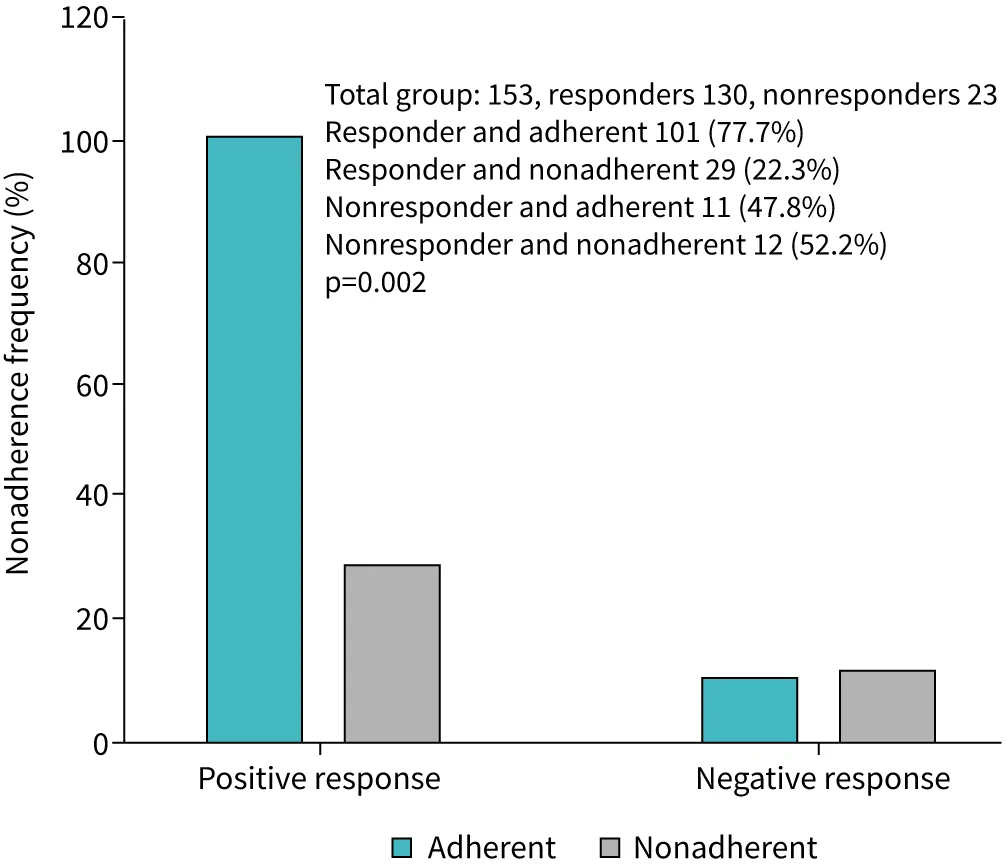
Cross tabulation of inhaled corticosteroid adherence as dichotomous trait (prescription possession ratio <75% equals nonadherence) in responder and nonresponder groups to mepolizumab.

We conducted comparisons of ICS adherent and nonadherent groups (regardless of mepolizumab response status) to explore impact of ICS adherence on overall patient outcomes (table 4). The only significant difference observed was the higher end of treatment median *F*_ENO_ (ppb) 57 (IQR 35, 81) *versus* 34 (IQR 21, 61), p=0.009 (figure 2) and higher highest *F*_ENO_ (ppb) 77 (IQR 51, 118) *versus* 53 (IQR 26, 88), p=0.04, in the nonadherent group than in the adherent patients.

**FIGURE 2:**
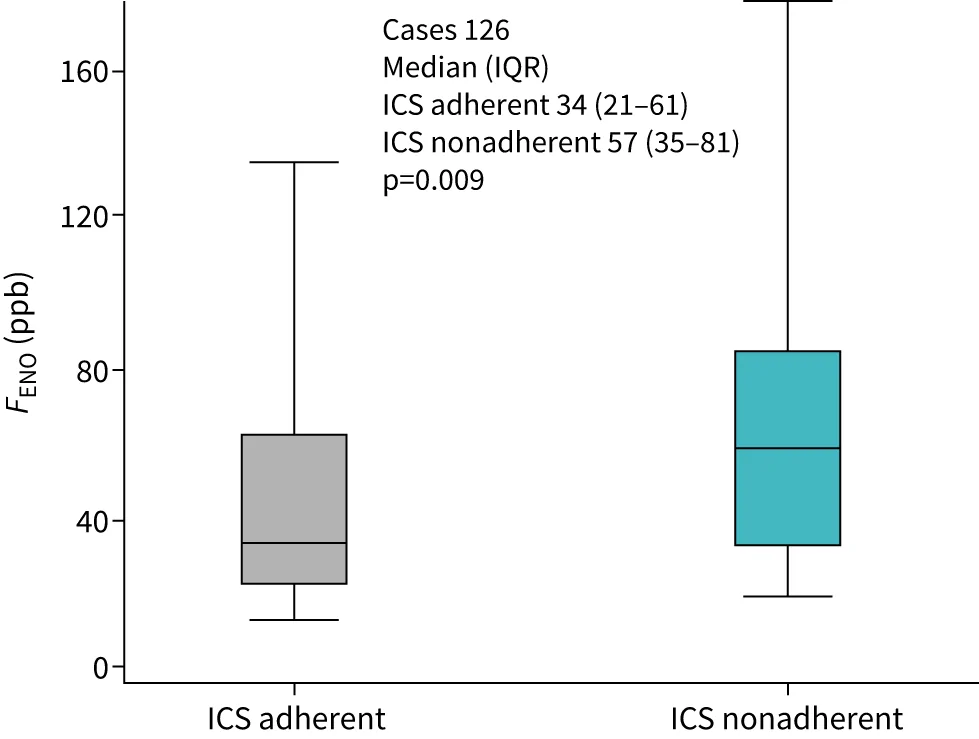
Median exhaled nitric oxide level in inhaled corticosteroids (ICS) adherent and nonadherent groups measured after 12 months of mepolizumab treatment. Groups compared using Kruskal–Wallis test. *F*_ENO_: exhaled nitric oxide fraction; IQR: interquartile range.

### Factors influencing ICS adherence during mepolizumab treatment

Logistic regression analysis was conducted with binary ICS adherence status during mepolizumab treatment (adherent, PPR ≥75%; nonadherent, PPR <75%) as the dependent variable. Independent variables included age, BMI, baseline PPR (continuous, measured over the 12 months before treatment) and the following continuous variables measured during mepolizumab treatment: exacerbation frequency, ACQ-6 score, blood eosinophil count and *F*_ENO_ (table 5). Baseline ICS adherence was the only significant predictor of ICS adherence during mepolizumab treatment in this analysis, with an odds ratio of 1.11, 95% CI 1.07–1.16, p<0.001. Correlation analysis between baseline PPR and PPR during mepolizumab treatment demonstrated a strong correlation (r=0.75, 95% CI 0.67–81, p<0.0001) (figure 3).

**TABLE 5:** Logistic regression analysis for inhaled corticosteroid (ICS) adherence during mepolizumab treatment using the dependent trait binary ICS adherence^#^

Variable	Coefficient	Standard error	Wald	Odds ratio	95% CI	p-value
**Age (years)**	0.06	1.07	0.30	1.00	0.96–1.05	0.7
**BMI (kg·m^−2^)**	−0.10	0.16	−0.86	0.96	0.90–1.07	0.3
**ICS adherence at baseline**	0.74	0.06	5.87	1.11	1.07–1.16	<0.001
**Asthma exacerbations after mepolizumab**	−0.35	0.49	−0.53	0.94	0.78–1.14	0.4
**A&E visits after mepolizumab**	0.21	1.38	0.20	1.07	0.54–2.1	0.8
**ACQ-6 score after mepolizumab**	−0.78	1.02	−1.47	0.71	0.45–1.16	0.1
**Highest eosinophils after mepolizumab**	−4.5	7.15	0.49	2.1	0.02–3.4	0.6
**Highest *F*_ENO_ after mepolizumab**	−0.01	0.02	−0.40	0.99	0.98–1.01	0.6

Logistic regression analysis for the dependent variable ICS adherence in the 12 months during mepolizumab treatment as a binary variable (ICS adherence PPR >75%) and the independent continuous variables of age, body mass index (BMI), ICS adherence pre-mepolizumab, asthma exacerbations, and during/end of mepolizumab treatment traits of accident and emergency (A&E) attendance, 6-item Asthma Control Questionnaire (ACQ-6) score, highest blood eosinophil count and highest exhaled nitric oxide fraction (*F*_ENO_) level. The sample size was 158 cases (117 ICS adherent patients and 41 ICS nonadherent). ^#^: prescription possession ratio (PPR) <75%=nonadherence state

**FIGURE 3:**
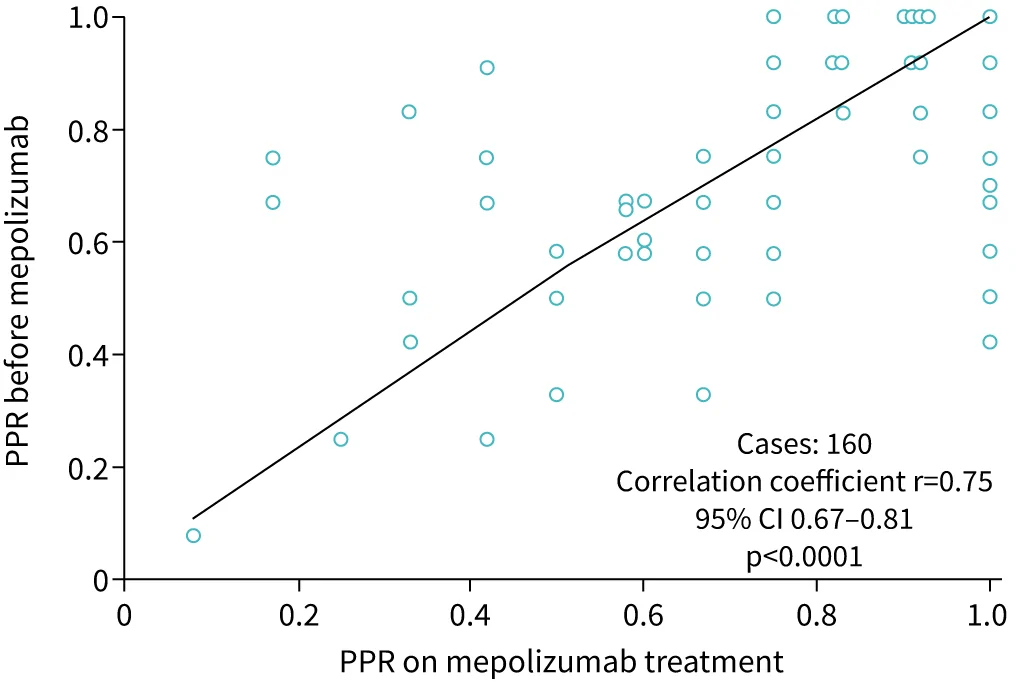
Correlation analysis of prescription possession ratio (PPR) as continuous variable over the 12 months before and the 12 months during mepolizumab treatment, demonstrating a close correlation in adherence status between the two periods.

## Discussion

This real**-**life retrospective study demonstrates the effectiveness of mepolizumab in treating severe eosinophilic asthma, with up to 80% achieving a positive response, which is in keeping with previous studies [4]. Our study confirmed the *a priori* hypothesis that ICS nonadherence whilst on mepolizumab treatment was more common in nonresponder participants. ICS nonadherence at baseline (the 12 months before mepolizumab initiation) was the only predictor of ICS nonadherence during mepolizumab treatment. Our findings support the notion that ICS adherence should be checked and optimised prior to and during mepolizumab treatment to improve treatment response [10].

Suboptimal ICS adherence remains challenging in severe asthma and is an important contributor to poor disease control [6, 19]. In our study, ICS nonadherence was significantly associated with a negative mepolizumab treatment response, was prevalent (30%) prior to biologic initiation and remained prevalent (26%) during mepolizumab treatment. These nonadherence rates were marginally lower than the rates of 35–59% reported in unselected severe asthma cohorts [5, 6, 19, 20]. However, optimised adherence is a prerequisite for mepolizumab initiation, and our practice includes detection and optimisation of ICS adherence prior to mepolizumab initiation, in accordance with UK NICE recommendations [17]. In theory, all patients should demonstrate optimised adherence to ICS prior to commencing biologic therapy; however, this is not true in real life, as reflected by the still signficant nonadherence rates observed in our study (26–30%) and other studies (ranging from 9 to 58%) [6, 21–24]. These findings indicate that a significant minority of cases would remain ICS-nonadherent even if they were on biologic treatment. Optimised adherence to ICS is challenging in clinical practice because of the limitations of currently available methods to detect it and the lack of effective tools to optimise it [19, 22]. Individuals with poor ICS adherence may still need to be treated despite their nonadherence if the harm associated with withholding biologic treatment is perceived to outweigh any benefit from waiting until ICS adherence can be optimised, which may never occur.

The only significant predictor of ICS nonadherence during treatment identified from the logistic regression analysis was the baseline “pre-mepolizumab” ICS nonadherence, which is in keeping with another study finding of similarity in ICS adherence rates before and after initiating mepolizumab [10]. Therefore, patients who are nonadherent before mepolizumab are likely to stay nonadherent during the treatment period and are more likely to have a negative treatment response. In the logistic regression analysis, *F*_ENO_ was not a significant predictor of ICS nonadherence, but *F*_ENO_ level was significantly higher in the ICS nonadherent group than that in the ICS adherent group. A high *F*_ENO_ level despite ICS treatment is a marker of ICS nonadherence status [11]. High *F*_ENO_ is also driven by other factors than ICS nonadherence, such as ICS resistance, leading to sub-optimal suppression of *F*_ENO_ despite adequate adherence to ICS [22, 23, 25]. High *F*_ENO_ is associated with an increased risk of exacerbations [13, 26] and, in the context of mepolizumab treatment, has been found to be related to asthma exacerbation phenotype (those with high *F*_ENO_ were more likely to express an eosinophilic exacerbation phenotype) [27]. ICS nonadherence during mepolizumab treatment response may contribute to a state of incomplete control of type 2 inflammation manifesting in inadequate suppression of eosinophil- and other IL-13 axis-mediated inflammation [28].

The association between raised *F*_ENO_ and a negative response to mepolizumab was not confirmed in our study, despite the observation of a nonsignificant trend towards higher *F*_ENO_ among mepolizumab nonresponders than responders. Other studies also did not a demonstrate link between high *F*_ENO_ and a negative mepolizumab response [14, 29, 30]. Therefore, despite the plausible hypothesis that high *F*_ENO_ would increase the chances of a poor or negative response to mepolizumab, this remained unconfirmed. *F*_ENO_ production in airway epithelium is IL-13 cytokine axis driven and is independent of the anti-IL-5 effect of mepolizumab, which is reflected in the modest or no effect of mepolizumab on *F*_ENO_ level [3, 31, 32]. The effective anti-eosinophilic action of mepolizumab was adequate to achieve a positive clinical response in the majority of cases (80%), but in a minority of cases, such effect was inadequate to suppress T2 inflammation (both *F*_ENO_ and eosinophilic), leading to a negative treatment response. Therefore, whilst high *F*_ENO_ may still be associated with a negative mepolizumab response, given the fact that negative responses occurred in a minority of cases, our study lacked the power to confirm or refute this notion.

Although only 45% of the eligible participants within our network were included in the analysis, the study sample is likely to be representative of the total eligible population, as we applied no inclusion restrictions apart from the availability of consent and treatment response outcomes. In addition, our study cohort characteristics are in keeping with those reported by the UK Severe Asthma Registry for patients with severe and difficult-to-treat asthma [21], in whom frequent exacerbations (median per annum: 6), mOCS use (49%), prior biologic treatment (15%) and multiple co-morbidities, dominated by obesity, GORD and chronic rhinosinusitis, were common. Despite these adversities, the positive response to mepolizumab was high (80%) and was manifested by significant reductions in exacerbation, mOCS use and emergency care attendance, and improved asthma control (those with an ACQ-6 score <1.5 increased from 11% at baseline to 38% after treatment) in keeping with previous reports [4]. Exploring predictors of a positive response to mepolizumab, we did not observe an effect of baseline blood eosinophil count on treatment response (allowing for prior selection of eosinophilic (≥0.3×10^9^·L^–1^) subjects as a treatment criterion), in keeping with previous studies [30, 31]. However, on-treatment eosinophil suppression was significantly greater in mepolizumab responders than nonresponders, implying that insufficient eosinophil suppression may drive a nonresponse to mepolizumab [29].

The effect of ICS nonadherence on other biologic treatments may differ from that observed with mepolizumab. A study of benralizumab response and ICS adherence reported that ICS nonadherence was not associated with a failed response after 6 months of treatment [24]. In contrast to mepolizumab, benralizumab triggers cell-mediated apoptosis, resulting in eosinophil depletion [33, 34] and leading to little or no residual type 2 inflammation in the airways of patients treated with benralizumab [15]. The SHAMAL study indicated that patients with severe eosinophilic asthma who are well controlled by benralizumab treatment could decrease the maintenance ICS dose without compromising asthma control. However, in a later study, upon reduction of the ICS dose, patients expressing high *F*_ENO_ fared less well, with declines in lung function and an increased risk of loss of asthma control [35]. In a real-life study, we also observed that a significant minority of patients with a poor response to benralizumab still exhibited airway eosinophilia, which was not always predicted by *F*_ENO_ levels [36]. Therefore, the effect of ICS nonadherence on benralizumab response remains uncertain. Optimising ICS adherence during benralizumab therapy may still be required to maintain asthma control and reduce the risk of exacerbation; however, down-titration of the ICS dose in those who achieve clinical remission of asthma would be prudent to reduce the side-effects associated with long-term high-dose ICS use [37]. The latter may also hold true for mepolizumab and other biologic treatments, as those who achieve a super-response or “clinical remission” are probably more likely to be able to reduce and even stop ICS treatment, with some patients self-stopping treatment if they perceive no benefit or no longer experience the regular asthma symptoms that prompt inhaler use [38]. In our study, the number of patients who met the clinical remission criteria was too small (13%) to allow for ICS adherence comparisons. A recent study of the effect of ICS nonadherence on exacerbations and asthma control in patients treated with tezepelumab reported no significant effect and argued for ICS dose down-titration especially in those achieving clinical remission [37]. Tezepelumab's broad effect on T2 inflammation (suppressing both eosinophils and *F*_ENO_) may make ICS use less influential on treatment response and exacerbation risk [39]. However, a significant number of patients treated with tezepelumab do continue to have an increased level of blood/sputum eosinophils and raised *F*_ENO_, and nonadherence or significant reduction in ICS dose in such cases may increase the likelihood of a poor treatment response. It is likely, therefore, that regardless of which biologic is being used, a personalised approach to ICS adherence and dose optimisation is needed that takes into account asthma remission status and T2 inflammation marker activity. This would allow some patients to reduce their dose to an anti-inflammatory reliever therapy regimen, whereas others with less well-controlled disease will require enhancement of ICS adherence and dose adjustment to match their clinical requirements.

### Strengths/limitations

This study offers valuable insights into the impact of ICS nonadherence on mepolizumab treatment response in patients with severe eosinophilic asthma. The study's limitations are related to its retrospective design, data incompleteness and reliance on PPR, which tends to overestimate adherence. However, its strengths include the use of real-world data, a clinically well-characterised cohort, a relatively large sample size and 12 months of follow-up data, providing sufficient power to identify the influence of ICS nonadherence on mepolizumab response. However, compared to the study power calculation, the negative response rate was lower than predicted, alongside a lower prevalence of ICS nonadherence, which would have reduced the study's power. Identification of an association between ICS nonadherence and a negative mepolizumab response despite reduced study power and allowing for the over-estimation of the adherence status due to the use of the PPR (*e.g.* a patient may collect a prescription but may still not use it) would strengthen this study's findings. Mepolizumab resulted in a significant reduction in the proportion of patients requiring mOCS; however, the proportion remained high (41%). Our registry did not distinguish between those needing mOCS for asthma and those needing it for adrenal insufficiency. This limitation may in part explain the relatively high proportion remaining on mOCS post-mepolizumab.

### Conclusion

Among severe eosinophilic asthma patients, ICS nonadherence during mepolizumab therapy was associated with mepolizumab nonresponse and raised *F*_ENO_ levels compared to good ICS adherence. Baseline ICS nonadherence strongly correlated with ICS nonadherence during mepolizumab treatment. ICS adherence influences mepolizumab treatment outcomes, prompting the need for effective strategies to detect and enhance ICS adherence to improve treatment response and patient outcomes.

## Data Availability

Data sharing is allowed subject to ethics clearance.
